# Shielded Coaxial Optrode Arrays for Neurophysiology

**DOI:** 10.3389/fnins.2016.00252

**Published:** 2016-06-09

**Authors:** Jeffrey R. Naughton, Timothy Connolly, Juan A. Varela, Jaclyn Lundberg, Michael J. Burns, Thomas C. Chiles, John P. Christianson, Michael J. Naughton

**Affiliations:** ^1^Department of Physics, Boston CollegeChestnut Hill, MA, USA; ^2^Department of Biology, Boston CollegeChestnut Hill, MA, USA; ^3^Department of Psychology, Boston CollegeChestnut Hill, MA, USA

**Keywords:** multielectrode array, extracellular, optogenetics, nanotechnology, neuroelectronic, optrode

## Abstract

Recent progress in the study of the brain has been greatly facilitated by the development of new tools capable of minimally-invasive, robust coupling to neuronal assemblies. Two prominent examples are the microelectrode array (MEA), which enables electrical signals from large numbers of neurons to be detected and spatiotemporally correlated, and optogenetics, which enables the electrical activity of cells to be controlled with light. In the former case, high spatial density is desirable but, as electrode arrays evolve toward higher density and thus smaller pitch, electrical crosstalk increases. In the latter, finer control over light input is desirable, to enable improved studies of neuroelectronic pathways emanating from specific cell stimulation. Here, we introduce a coaxial electrode architecture that is uniquely suited to address these issues, as it can simultaneously be utilized as an optical waveguide and a shielded electrode in dense arrays. Using optogenetically-transfected cells on a coaxial MEA, we demonstrate the utility of the architecture by recording cellular currents evoked from optical stimulation. We also show the capability for network recording by radiating an area of seven individually-addressed coaxial electrode regions with cultured cells covering a section of the extent.

## Introduction

A major goal of neurophysiology is to understand how ensembles of neurons generate, store and recall representations of the physical world and coordinate responses to its changing environment. To understand these fundamental capacities, neuroscientists investigate the electrical activity of neurons in individual and networked form to correlate patterns of activity to specific behaviors or cognitions. To this end, some of the goals of neural device development are to increase biocompatibility; to increase the recording scale, i.e., the ability to record and stimulate hundreds to thousands of individual neurons simultaneously without compromising cell viability; to increase the duration of electronic coupling to neurons over extended periods of time (days to months); and to better dissociate the many neurophysiological events (action potentials, excitatory/inhibitory post-synaptic potentials, *etc*.) that occur in a neural circuit. Many years of device development and refinement have produced state-of-the-art tools capable of measuring action potentials (APs) originating from multiple neurons, as well as tracking propagation of APs (Blanche et al., 2005; Bakkum et al., 2013; Buzsáki et al., 2015). One such tool is the microelectrode array (MEA), which is highly scalable and able to be utilized in a multiplex assay, the type necessary to study ensembles of neurons (Hierlemann et al., 2011).

Well-characterized and commercially-available MEAs fall under two categories: *in vitro* arrays, consisting of planar metal microelectrodes^1^, and *in vivo* arrays, which can vary from 2D (Michigan array; Najafi and Wise, 1986; Seymour and Kipke, 2007) and 3D (Utah array; Maynard et al., 1997) structures to flexible polymer devices (Rodger et al., 2008), with electrode separations from several tens to several hundreds of microns. In considering ways to further advance extracellular recording, one approach is to decrease the scale of the recording element from the micro- to the nanoscale. Next generation versions of MEAs (Spira and Hai, 2013) include nanowire electrode arrays (Robinson et al., 2012), field effect transistor arrays (Voelker and Fromherz, 2005; Duan et al., 2012), novel structure arrays (Hai et al., 2009) and nanopillar arrays (Xie et al., 2012; Lin et al., 2014). In some cases, such technologies have brought the electrode pitch down to the 20 micron range (Hutzler et al., 2006; Frey et al., 2010).

Although recent advances have reduced electrode scale and pitch, a prevailing problem in extracellular recording from neuronal networks is the ability to identify the individual neurons from the local field potentials (LFPs) recorded by one or more adjacent electrodes, a process known as spike sorting. Even with high density MEAs, synchronous discharges of similar waveforms from multiple neurons equidistant from a recording site make spike sorting difficult (Nadasdy et al., 1998). Complexities in neuronal firing modes, neuronal morphology and other intrinsic properties all complicate the separation of neurons based on the recorded extracellular field potential waveforms (Einevoll et al., 2012; Buzsáki et al., 2015). The development of validated spike sorting algorithms and a desire for standardization has been previously discussed, yet the process depends on subjective standards and time-consuming offline data analysis (Einevoll et al., 2012; Obien et al., 2014). The need for spike sorting is a direct result of the phenomenon of electrical crosstalk, wherein an electrical signal sourced near one electrode is also sensed by one or more neighboring electrodes. Crosstalk makes spatiotemporal identification of a signal source difficult, even with offline spike sorting. Unfortunately, reducing the pitch and scale of conventional electrodes has only magnified the problems associated with crosstalk.

Another possibility for electrode development is the integration of optical components with electrodes, producing devices called “optrodes.” Optrodes (Zhang et al., 2009) enable electric field sensing simultaneous to local light delivery and so provide a closed-circuit interface to light-sensitive proteins and light-emitting biosensors such as channel rhodopsins and genetically-encoded calcium indicators, respectively (e.g., optogenetics). These advances in bioengineering now permit actuation and sensing of individual or groups of neurons depending upon their phenotype and anatomy, among other factors (see Boyden et al., 2005; Deisseroth, 2010, for reviews). Thus, optogenetic tools overcome a limit of conventional extracellular recording from neuronal networks, which do not permit precise electrical actuation of a specific cell type within an assembly of multiple neuronal types (Butovas and Schwarz, 2003). As such, hybridization of optical and electrical elements into optrode arrays can help in the progression of traditional MEA technology for use with optogenetics (Zhang et al., 2009; Kim et al., 2013; Wu et al., 2015). Nonetheless, the technical issues of electrical crosstalk in MEAs, and local light delivery in optogenetics, have not been fully resolved, such that new approaches are needed to facilitate the targeting of specific cell types within a neuronal assembly.

Here, we present a shielded electrode architecture that can both reduce crosstalk and integrate optical stimulation. The shielded electrode has a coaxial architecture that consists of two concentric metals in a vertically-oriented cylindrical structure, separated by an electrically-insulating layer. The inner metal is a micro/nanowire that acts as a coax core, while the outer metal functions as a shield, in a manner similar to a macroscale radio frequency coaxial cable, such as that used for cable TV. As mentioned, crosstalk between pixels of conventional devices with high spatial resolution is a consequence of their unshielded nature; a shielded coaxial device can suppress this limitation, uniquely allowing increases in pixel density. Also similar to that macroscale coax is the micro- and nanoscale version's ability to propagate subwavelength electromagnetic radiation, including visible light (Rybczynski et al., 2007; Merlo et al., 2014). Nanoscale coaxial arrays have been previously used by some of the present authors (Rizal et al., 2015) in a variety of biological (Archibald et al., 2015), chemical (Zhao et al., 2012; Rizal et al., 2013), optical (Rybczynski et al., 2007; Merlo et al., 2014) and photovoltaic (Naughton et al., 2010) devices. Additionally, the principle of a single coaxial structure as an optrode was validated through the use of a tapered, metal-coated optical fiber for studies in non-human primates (Ozden et al., 2013). In this article, we provide proof of principle that a multiplexed nanoscale coaxial optrode can lead to a next generation of optrode neurointerfaces capable of very high spatial resolution electrical sensing and local optical stimulation.

## Materials and methods

### Simulation of device function

**Figure 1:**
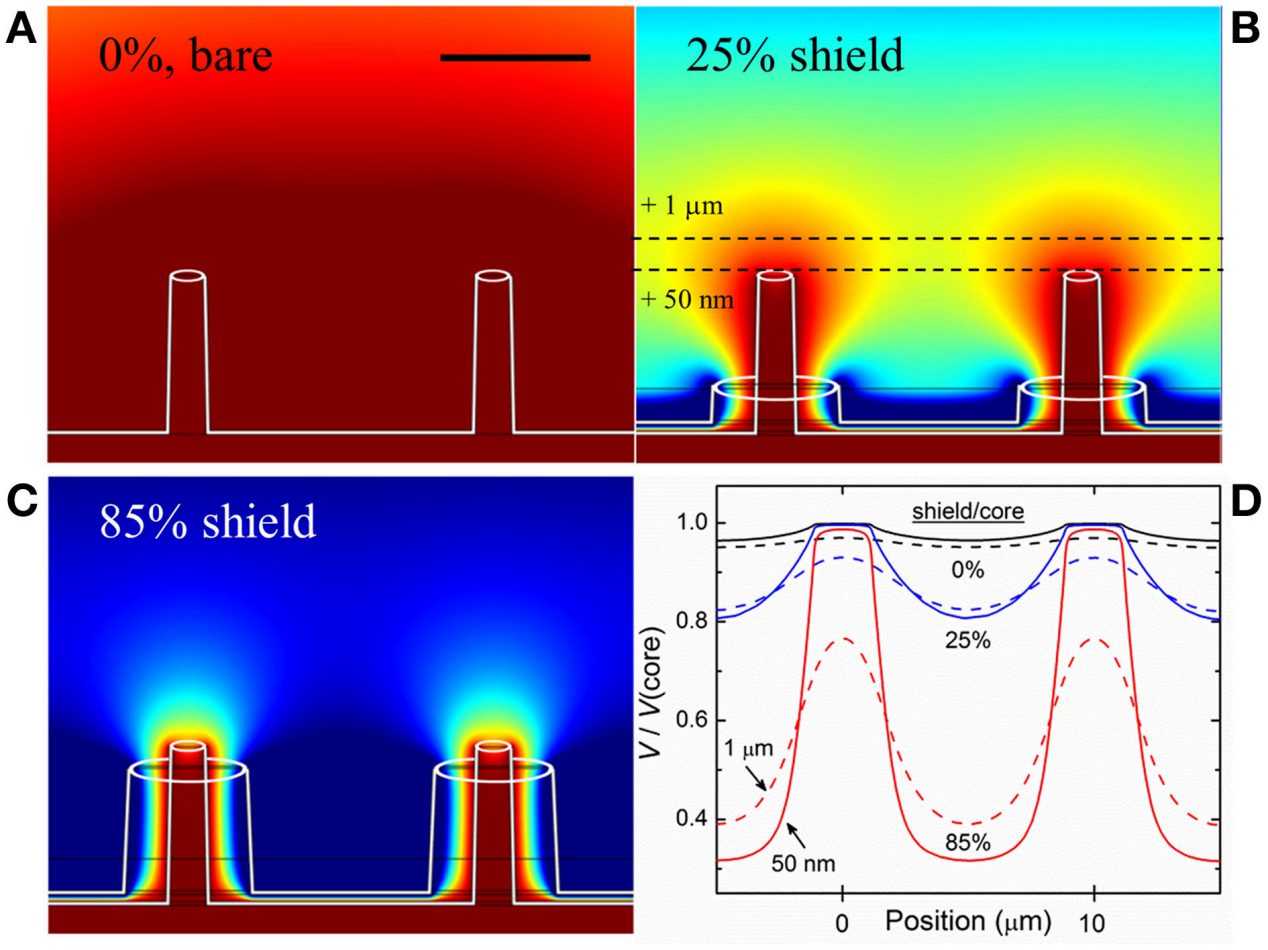
**Simulation of electric potential profile. (A)** Equipotential contours for bare (unshielded) electrodes, 5 μm tall and 10 μm apart, biased at 100 μV (ground at infinity). Scale bar: 5 μm. **(B)** Electrodes with ground shield 25% the height of the biased core (1.25 μm). **(C)** Electrodes with shield 85% the height of the biased core (4.25 μm). Dark red represents areas where >95% of the signal from a source (e.g., action potential/neuron spike) would be seen by the electrode while dark blue represents areas where <20% of the signal would be seen. As the shield progresses in height, overlapping areas shrink and result in discretized electrodes, and thus reduced electrical crosstalk. **(D)** Plots of electric potential vs. lateral position for the three cases shown, for two constant heights above the core tips, 50 nm and 1 μm, and scaled to the core potential, further demonstrating the virtue of the shielded architecture: bare electrodes only negligibly resolve the spatial variation of *V/V*(core), while the shielded coaxes in **(C)** show clear discrimination.

A computational model of the device, intended to simulate the environment in which a neuron is in close proximity to multiple electrodes, was made using the finite element method (FEM) simulation software COMSOL Multiphysics, employing realistic materials parameters. A hexagonal pattern of coaxial electrodes was placed in an electrolyte solution (having the same electrical properties as the medium used in experiment, i.e., dielectric constant ε ~ 80, electrical conductivity σ ~ 1.5 S/m). Although crosstalk and the detection of field potentials *in situ* is influenced by myriad factors including cell type, distance from electrode and the nature of the contact with electrodes, the purpose of this simulation was to find the amplitude of the potential at the recording electrode surface generated by a source (e.g., neuron spike) as a function of separation distance. Green-Lorentz reciprocity (Lorentz, 1896) reduces this problem to solving Poisson's equation for the scalar potential generated from the recording electrode as a voltage source. The simulations, shown in Figure 1, were performed for non-shielded electrodes (Figure 1A), coaxial electrodes with an outer shield electrode comprising 25% of the inner (recording) electrode height (Figure 1B), and coaxial electrodes with a shield comprising 85% of the inner electrode height (Figure 1C).

### Device fabrication

**Figure 2:**
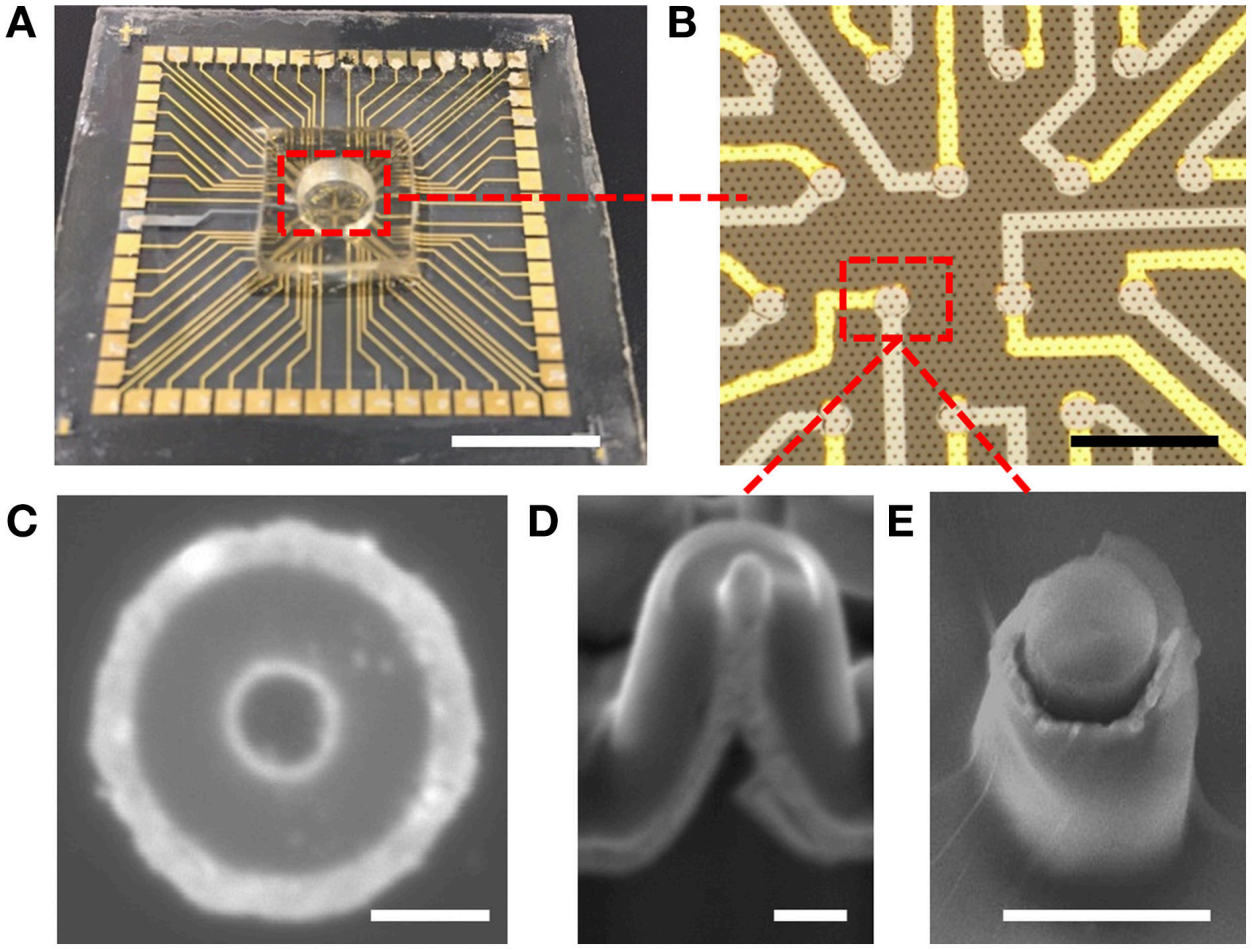
**Coaxial electrode array. (A)** Coaxial microelectrode array (cMEA) on glass substrate. Scale bar: 10 mm. **(B)** Optical micrograph showing cMEA sensing areas. Gray lines are Cr (shield) address lines, yellow lines are Au (core) address lines, and circular overlapping areas are the coaxial sensing areas. Small dots throughout image are the underlying pillar array. Scale bar: 100 μm. **(C)** Top view SEM image of a single coax in a coaxial nanoelectrode array (cNEA). Scale bar: 200 nm **(D)**, **(E)** 30° tilted view SEM images of nanoelectrode (scale bar: 200 nm) and microelectrode (scale bar: 2 μm) coaxial array architectures, respectively.

Devices were fabricated on either borosilicate glass or Si substrates, both pre-cut to dimensions necessary for compatibility with an amplifier system. Two different pitch and pillar sizes were prepared, one for coaxial nanoelectrode arrays (cNEA) and one for coaxial microelectrode arrays (cMEA). The Si substrate was used for the cNEA fabrication and was patterned and etched to contain a 200 mm^2^ pillar area containing 200 nm diameter × 2 μm tall pillars at 1.3 μm hexagonal pitch. For the cMEA, a 100 mm^2^ area containing an SU-8 polymer nanopillar array (2 μm diameter × 5 μm tall pillars at 10 μm hexagonal pitch) was fabricated using nanoimprint lithography (NIL), similar to previously published work (Rizal et al., 2013). Standard contact photolithography was used to generate subarrays containing a fixed number of pillars. Coaxial electrodes were then prepared by sequential metal, dielectric and metal coatings onto the pillars, yielding the structures shown in Figure 2. In order to prepare such devices for neuroelectronic recording and stimulation, the inner coaxial (core) electrode must be exposed to permit physical access to neurons in proximity to the sensing element. We achieved this by mechanically polishing the array, thereby “decapitating” the structures, to leave behind the open-ended microscale coaxial electrodes shown in Figures 2C–E. To facilitate this polishing, a polymer film (SU-8) was first spin-coated over the array and hardened, mechanically stabilizing the structure. Devices with the SU-8 core, which is optically transparent, can be further prepared for opto-neuroelectronic studies by additional polishing to ensure that the core tops are metal-free (see Figure 2C). Subsequent selective etching of the outer shield and annulus can then be performed to expose a greater core metal electrode surface area. Figures 2A,B show optical micrographs of a completed extracellular interface array device. The coax inner (core) and outer (shield) conductors are sputtered Ti:Au (10 nm: 110 nm thickness) and Cr (110 nm), respectively, and the dielectric is 150 nm thick atomic layer-deposited Al_2_O_3_. The coaxial sensing regions of the cNEA were 50 μm in diameter, each region containing ~1300 individual coaxes, while the subarrays in the cMEA (used for optogenetic studies in this paper) were 20 μm in diameter and contained 8 ± 1 individual coaxes.

### Leech ganglion extraction process

We recorded electrical transients using neurons dissected from a live specimen of the invertebrate medicinal leech *Hirudo Medicinalis*, in accordance with policies regarding the humane use of invertebrate animals in research at Boston College. The leech was anesthetized by placement in a 5:1 H_2_O:C_2_H_6_O solution for 10 min and afterwards, pinned to a dissection tray while submerged in a phosphate buffered saline solution. An incision was made on the dorsal side of the specimen and the skin was pinned to the side and muscle tissues were removed to expose the nerve cord. Starting with the 4th ganglion from the head, the nerve cord was isolated by removing the coating tissue (of the nerve cord) as well as the underlying skin. This was continued until the 18th ganglion (head and tail ganglion were left unexposed for pinning purposes); leaving 14 exposed ganglion sacs. The myelin sheath coating each individual ganglion sac was then removed in order to expose the ~400 neurons present in each sac. The nerve cord was then inverted and pinned on top of our device (the desheathed ganglion sac placed directly over a coaxial sensing region).

### HEK-293 cell preparation

Optically evoked field potentials were detected using human embryonic kidney cells (HEK-293) transfected with the blue-light sensitive channelrhodopsin ChR2(H134R) (Zhang et al., 2007). HEK-293 cells are similar in size to small neurons (~15 μm diameter) and do not natively express light sensitive ion channels, which make them a suitable heterologous expression system in the development and validation of novel optogenetic device interfaces. Our approach was similar to several prior reports (Lin et al., 2009; AzimiHashemi et al., 2014) in which HEK-293 cells were made to express ChR2, which mediated depolarizing, inward currents in response to blue light. Here, HEK-293 cells were cultured in Dulbecco's modification of Eagle's medium (DMEM) containing 10% fetal bovine serum (FBS) and 1% PenStrep Antibiotic in a 6 well culture dish. The pcDNA3.1/hChR2(H134R)-EYFP plasmid (#20940, Addgene, Cambridge, MA) was transfected into HEK-293 cells using Lipofectamine 2000 (Invitrogen) according to the user manual. In brief, ~4 μg of plasmid and 10 μl of Lipofectamine were transfected into HEK-293 cells. At 16 h post-transfection, the cells were transferred to a 6 well plate and grown in DMEM 10% FBS media supplemented with 500 μg/ml Geneticin (G418). Cells were cultured under G418 selection for ~2 weeks to obtain cultures of ~100% EYFP-expressing cells. A high percentage of EYFP-expressing HEK-293 cells were observed upon culturing the cells in the presence or absence of G418 in the media, suggesting the plasmid had stably integrated. After 2 weeks, the cells were cultured in DMEM media containing 250 μg/ml G418 to maintain a stable ChR2-EYFP expressing cell population (HEK-ChR2 cells).

To adhere cells to the coaxial structures contained in a teflon well (~3 cm diameter), coax devices were incubated in a sterile solution of 0.01% poly-l-lysine overnight at 37°C 5% CO_2_. HEK-ChR2 cells were trypsinized from cell culture dishes and recovered by centrifugation at 595 g for 6 min at 4°C. The cells were re-suspended in DMEM 10% FBS media containing 250 μg/ml G418 at a density of 1 × 10^6^ cells/ ml. A 0.1 ml aliquot of cells was added to one well of a coaxial device and cultured overnight at 37°C 5% CO_2_. The seeding density of cells almost completely covered the coaxial structures within 24–48 h of subsequent cell culture and adherence.

### Electrophysiology

Extracellular field potentials from the ganglion sac of a leech were recorded using the cNEA. Data were sampled at 25 kHz and amplified 200× using an SR560 low noise preamplifier (Stanford Research Systems, Inc.) and a Multiclamp 700B with Digidata 1440A (Molecular Devices, LLC) data acquisition system, and filtered using 30 Hz–30 kHz band pass filter. Events from transfected HEK-293 cells were recorded using a USB-MEA1060 60 channel amplifier, DAQ and MC_Rack software (Multi Channel Systems MCS GmbH). Coaxial MEA chips were fabricated to be compatible with this amplifier system, coated with poly-l-lysine and sat overnight prior to dispensing the cell culture onto the device. The measured peak-to-peak noise level of the device was (10 ± 4) μV, which was on the same order as our estimated value. We used the Johnson-Nyquist formula to calculate the intrinsic thermal noise level, $\delta \text{V}=\;\sqrt{4{\text{k}}_{\text{B}}\text{T}∕\text{C }}$ with $\;C=\;\frac{2\pi \varepsilon \text{L}}{\text{ln}\left(\text{b}∕\text{a}\right)}$, which yielded δV = 6.4 mV.

### Optical stimulation

A 473 nm DPSS laser (Model BL473-100FC ADR-700A, Shanghai Laser and Optics Century Co., Ltd.) coupled to a multimode 200 μm diameter optical fiber (Thor Labs) with a spot size of ~350 μm diameter was used for photo stimulation. The laser was triggered using a TTL signal (Stimulus Generator STG4002, Multichannel Systems) with a 1 s square wave pulse. In our first preparation, the tip of the optical fiber was positioned directly above the cMEA after plating with HEK-ChR2 cells. The tip of the optical fiber was initially fixed in a specific position over the array, actuated for 1 s (power 20 mW/cm^2^), and then repositioned using a micromanipulator before being actuated again. Throughout this illuminate-position-illuminate scanning sequence, all 30 available channels were monitored for light-evoked potentials. Upon event detection, a dose-response test was performed in order to characterize the sensitivity of each individual coaxial sensing region using a range of power settings from 0.5 to 30 mW/cm^2^. Optical power was established with a commercially available power meter according to the manufacture's instructions (Model 1916-R, Newport Corp.). In the second preparation, the same scanning sequence was used but the optical fiber tip was placed underneath the cMEA substrate to achieve optical illumination through the transparent SU-8 coax cores.

## Results

### Simulation of coaxial electrode arrays

To estimate the coaxial electrode array's spatial recording field, we performed 3D electrostatic modeling of an array of coaxial electrodes using FEM analysis. The device was modeled with the inner metal at a fixed potential (100 μV) and the outer metal at ground (reference), placed in a conducting solution (conductivity determined by particular medium used in experiment, described in Materials and Methods). From the simulations, we were able to generate profiles of the recording field surrounding the electrodes. 2D cross-sections of the profiles are shown in Figure 1 for coaxes having 5 μm core height and 10 μm array pitch. Keeping the core height constant, we simulated various shield heights (Figures 1B,C) and compared the results to the case of bare electrodes (i.e., no shield, Figure 1A). It is clear that as the shield height becomes closer to that of the core, the recording field spatial localization improves. Comparing the overlapping profile regions in each of the regimes shown (bare electrode, 25% shield height, 85% shield height), it can be seen that the field near bare electrodes overlaps that of its neighbors, while this overlap is suppressed for shielded electrodes. In other words, locally-shielded electrodes suppress electrical crosstalk. By approximating the proximity of an electrogenic cell to our electrode array to be 50 nm (Fromherz, 2003a), we were able to obtain a range of shield heights appropriate for sensitive extracellular AP recording and crosstalk suppression. The results of the simulations can be quantified by plotting the fraction of the electric potential of the core (e.g., 100 μV) that would be sensed certain distances from the core. Figure 1D shows calculations of this proportion, *V*/*V*(core), for two heights above the cores, 50 nm (solid lines) and 1 μm (dashed lines), for the three cases of Figures 1A–C, plotted along a horizontal distance. At 50 nm height, *V* above a core (i.e., Position ~0 or 10 μm) and *V* between cores (Position ~5 μm) differ by only 3% for the bare electrodes, but by more than a factor of 3 for the 85% shielded coaxes. At 1 μm height, the bare electrodes differ by <2%, and the 85% shielded coaxes by ~100% (i.e., a factor of 2), for these Positions. The goal of these simulations is to demonstrate the virtue of shielding for future MEA devices with closely-spaced electrodes, closer than typically exists in conventional MEA devices. Similar simulations were done for smaller, nanoscale coaxes, with comparable results, confirming that the shielding discussed here improves pixel discretization at all scales.

### Fabrication and characterization of coaxial nanoelectrode arrays (cNEA) and coaxial microelectrode arrays (cMEA)

**Figure 3:**
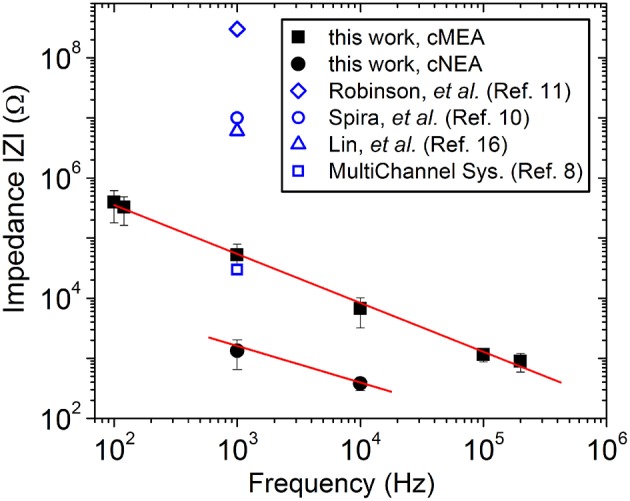
**Characterization of device**. Impedance measured as a function of frequency for an individual coaxial sensing region for the cMEA (solid squares) and the cNEA (solid circles). Lines are guides to the eye. Related devices found in the literature are included for comparison.

Nanocoax arrays were used to achieve high electrode density for proof of principle extracellular recordings from leech ganglion sacs, whereas microcoax arrays were fabricated at a more relaxed pixel density and larger diameter for testing the architecture as optrodes. Both designs used the same material thicknesses (see Materials and Methods). The materials used were chosen for their biocompatibility, as studied previously (Hassler et al., 2011; Kim et al., 2014). After deposition and etching steps (see Materials and Methods), scanning electron micrographs were taken (Figure 2) to confirm the core metal electrode was extended above the annulus and outer metal layer. To characterize the devices, DC resistance (between the inner and outer electrode) measurements were made first in air to verify device integrity (not shorted), with typical resistances in the GΩ range, as anticipated. A capacitance bridge was also used to measure the capacitance of the devices, and the measured values were on the scale of the calculated value based on geometry and material parameters. Electrochemical impedance measurements were then made across a 100 Hz–200 kHz frequency range. Many neurophysiological phenomena occur within the 0.1–10 kHz frequency band and, therefore, a low impedance value within this range is desired (Buzsáki et al., 2012). Both the cMEA and cNEA devices compared favorably to similar devices found in the literature, as well as commercial microelectrode arrays, Figure 3. The cMEA device had a higher impedance (|Z| = 52.9 ± 26.4 kΩ) than the cNEA (|Z| = 1.5 ± 0.7 kΩ) at 1 kHz, due to the latter having more coaxial pillars per coaxial sensing region and therefore more total electrode surface area (roughly 20 times more). Increased surface area of the 3D coaxial architecture is also the reason the impedances of our devices are lower than the other technologies represented in Figure 3.

### Recording of extracellular action potentials

**Figure 4:**
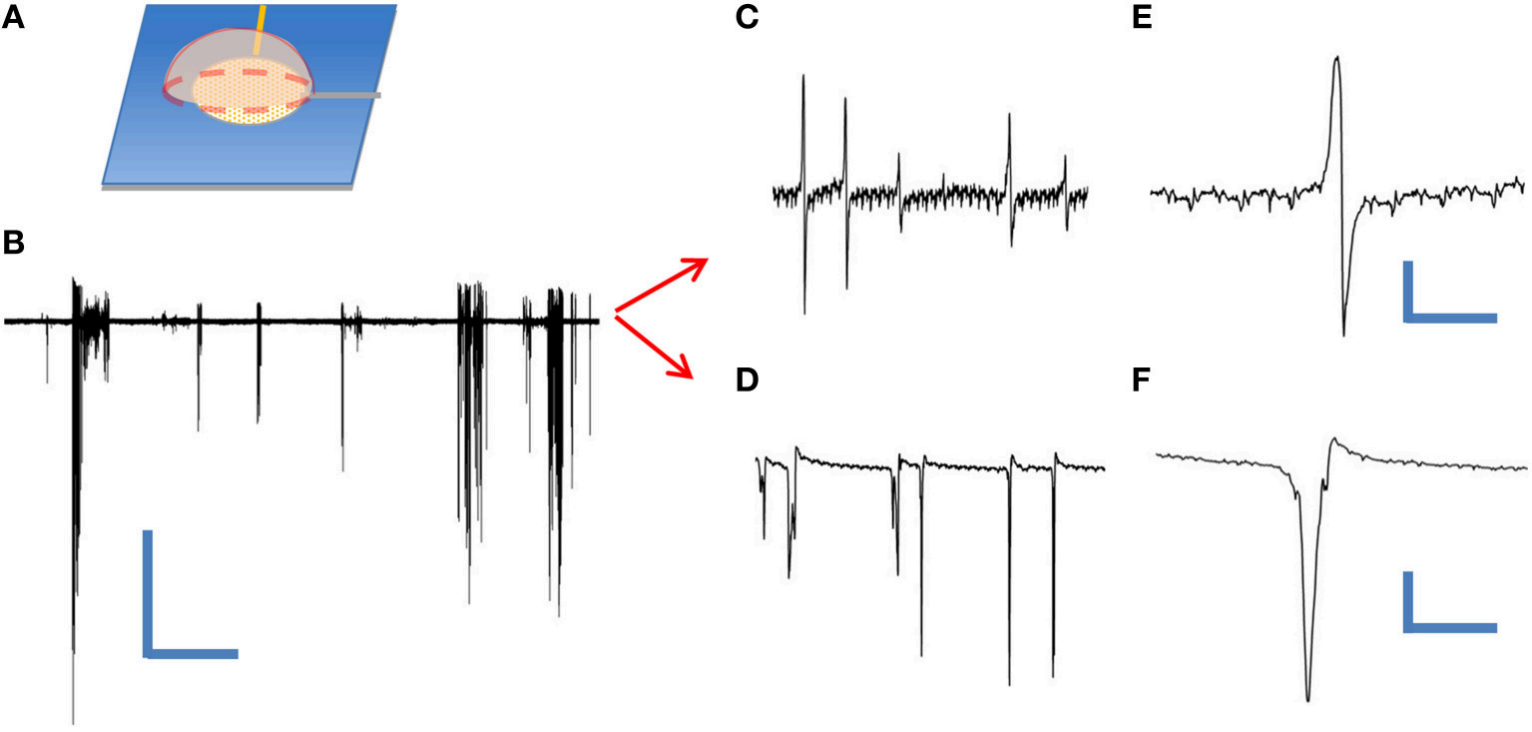
**Extracellular recording of dissociated leech neurons mechanically placed on top of coaxial sensing region of a cNEA. (A)** Schematic of ganglion sac placement onto an individual sensing region within the device. **(B)** Spontaneous bursts during 60 s recording. Scale bars: 400 μV/10 s **(C)** One waveform type found within burst. **(D)** Second waveform resembling extracellular action potential found during post-recording spike sorting analysis. **(E,F)** Closer looks at two distinct waveforms extracted during post-analysis spike sorting. Scale bars, upper right: 50 μV/10 ms, lower right: 200 μV/3 ms.

In order to test the utility of our device as an extracellular neuroelectronic sensor, we passively recorded from leech neuronal assemblies contained within an individual ganglion sac (Muller et al., 1981) using a cNEA device. We initially tested the condition of the cells by performing sharp electrode recordings from Retzius and N-cell types (contained within the same ganglion sac) and both showed typical waveforms (not shown) for such cells, as found in the literature (Muller et al., 1981; Fromherz, 2003b). Next, a different ganglion sac was chosen, desheathed and placed on top of a cNEA sensing region 50 μm in diameter containing ~1300 nanocoaxes (as depicted in Figure 4A). A weighted polymer mold was placed on the backside of the sac in order to promote electronic coupling (contact) with the electrode array. Multiple spontaneous activity bursts were clearly seen over a recording time of 5 min (Figure 4B) with a 10 kHz sampling rate. The experiment was repeated several times, each with a different neuronal assembly, with spontaneous bursts seen each time. Events were considered as anything reaching a threshold of 3 times the peak-to-peak noise level (noise ~ 10 μV). Post-waveform data analysis was performed and produced two unique waveforms (Figures 4C,E and Figures 4D,F), as seen in previous works, showing successful extracellular recording (Fromherz et al., 1991; Fromherz, 2003b).

### Optically-evoked potential deflections from HEK-ChR2 cells

**Figure 5:**
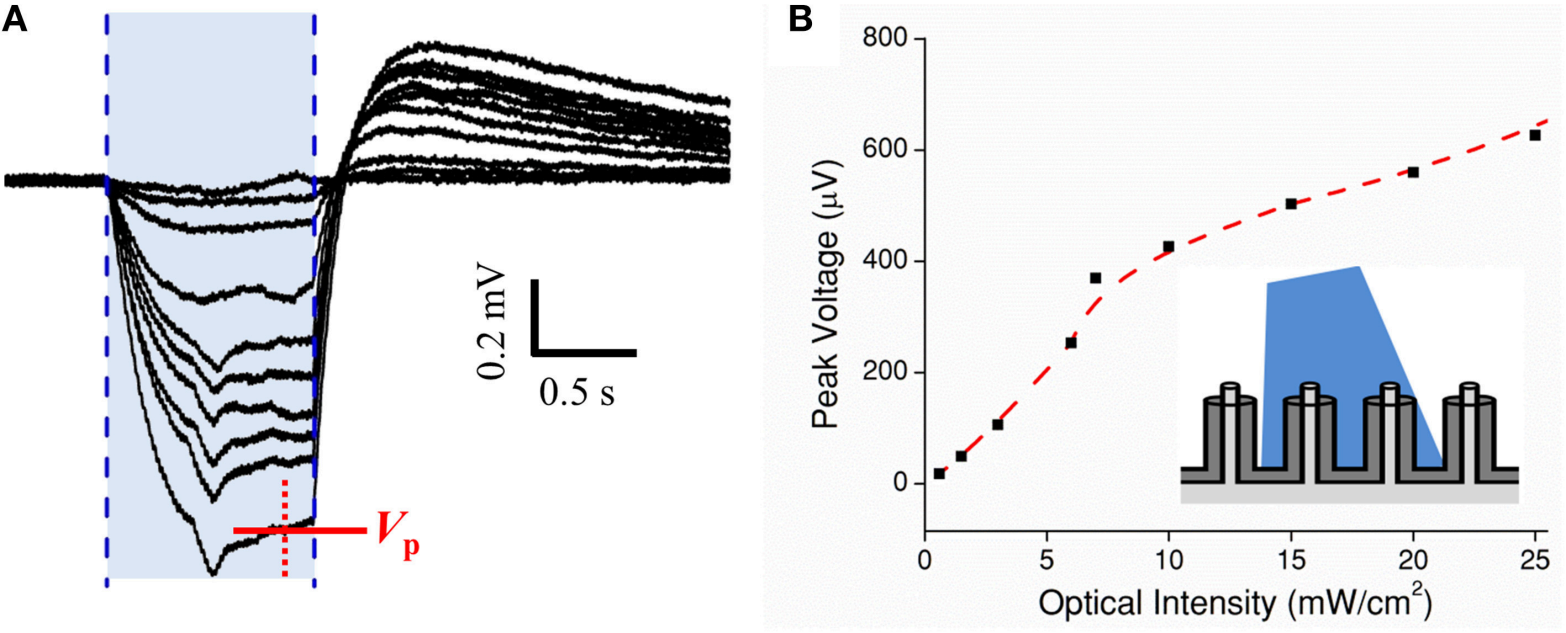
**Dose test of optogenetic HEK-ChR2 cells cultured onto a cMEA. (A)** Dose test during top side illumination (473 nm) of HEK-ChR2 cells cultured onto a cMEA. The shaded blue region indicates the light-on times and the red arrow indicates the time at which peak voltage was determined (signal having reached a local steady state). **(B)** Peak voltage as a function of power density with parametrically fitted line to guide the eye. Inset depicts light-from-above configuration.

Next, a cMEA was used to record current transients from HEK-ChR2 cells. The recording electrode consisted of a 5 × 6 array of individually-addressed coaxial sensing regions spaced 100 μm apart. Each 20 μm diameter sensing region contained 8 coaxes wired in parallel (i.e., all center conductors connected to each other, and all outer grounds connected to each other). Initially, the cMEA was scanned for light-evoked ChR2 potentials which appeared as negative deflections in the extracellular field potentials. Once successful event detection sites were found, a dose response test was performed by fixing the optical fiber tip directly above a particular sensing region under study and varying its intensity from 0 to 30 mW/cm^2^ in 2 mW/cm^2^ steps, Figure 5A. The response magnitude varied slightly (~20%) among regions tested. Each showed a characteristic spike upon initial stimulation (in response to cellular depolarization) before reaching a steady state followed by an after-potential once the laser was turned off. The after-potential is most likely due to the delayed rectifying K_v_ channels native to HEK-293 cells (Jiang et al., 2002). Figure 5B shows the peak voltage *V*_P_ recorded as a function of light intensity. The data show a response of ~50 mV/(mW/cm^2^) at low optical power, deceasing to ~10 mV/(mW/cm^2^) at higher power. In subsequent tests, light-evoked field potentials were evident at intensities as low as 0.5 mW/cm^2^. Cell coverage was confirmed by epifluorescence microscopy in ~40% of the regions within the 5 × 6 array. Importantly, a response to light stimulation was found in only in the regions with HEK-ChR2 cells, and not in those without cell coverage. Although the bandgap of the material in the coax annuli, Al_2_O_3_, is too large to generate electric current from visible light (such as could occur in a CMOS device with a lower bandgap material, Frey et al., 2010), we nonetheless performed the scanning procedure as described on a cMEA containing only cell culture media. In no case were potentials evident above the level of the intrinsic noise; therefore, the observed light evoked field potentials observed in the presence of HEK-ChR2 cells should reflect only the changes in the local electrical fields caused by ionic conductance in the light-gated ChR2 channels and are not contaminated by an artifact of photo stimulation *per se*.

**Figure 6:**
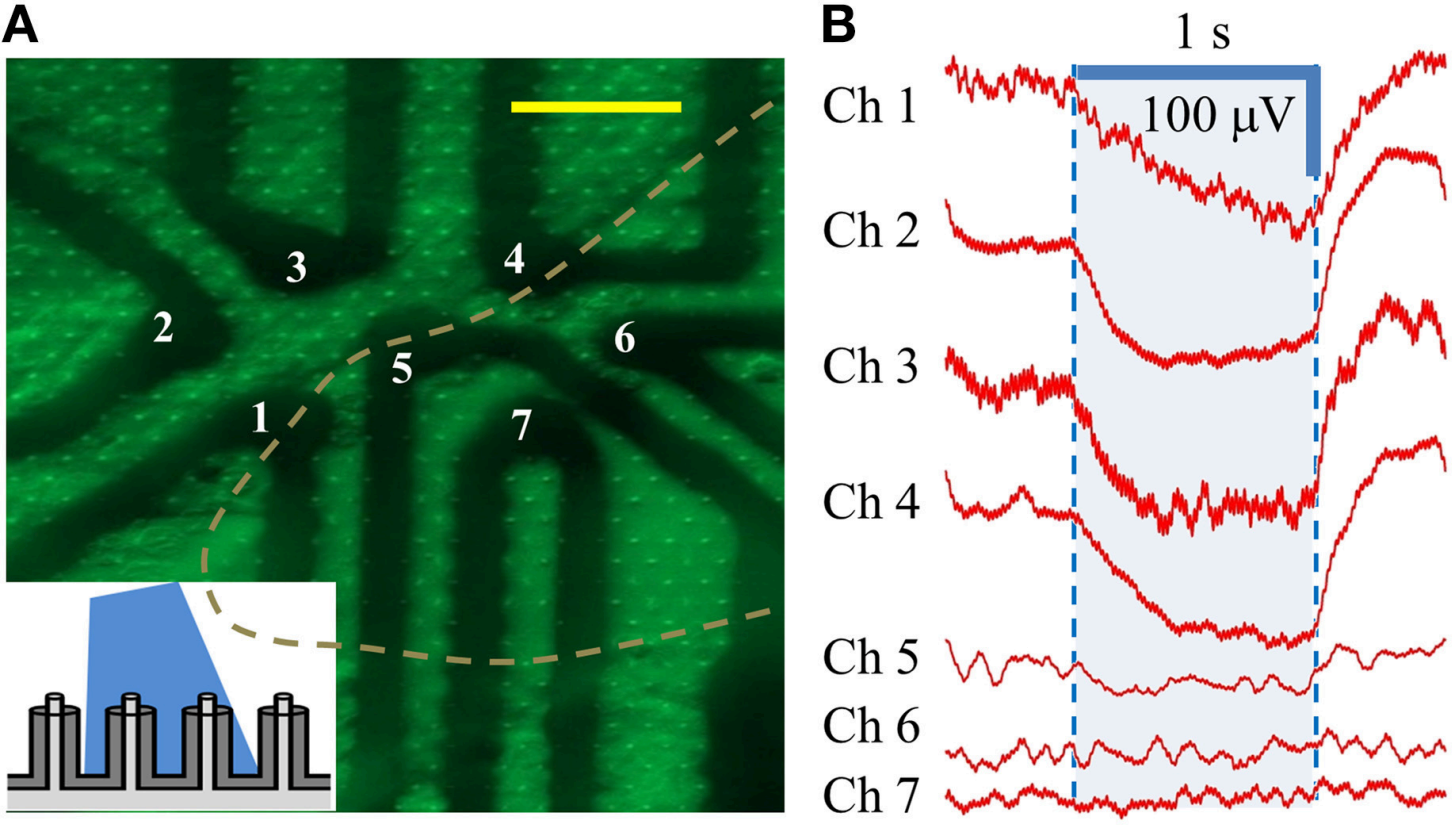
**Individually-addressed coaxial sensing regions in cMEA. (A)** Fluorescent microscope image of HEK-ChR2 cells covering a portion (in area left of dashed line) of 7 individually-addressed coaxial regions, each containing 8 coaxes. Inset depicts light-from-above configuration. Scale bar: 50 μm. **(B)** Electrical response (changes in LFP) of HEK-ChR2 cells to optical stimulation in the 7 sensing regions (473 nm wavelength; 20 mW/cm^2^). Shaded region denotes light-on times.

Our next experiment was performed on a cMEA containing 7 individually-wired, 20 μm diameter, coaxial sensing regions (again, with ~8 coaxes wired in parallel per region) spaced at a 60 μm pitch (i.e., center-to-center). One such area was imaged by epifluorescence in order to determine the cell coverage, as shown in Figure 6A. This image revealed 4 of the 7 sensing regions (those left of the dashed line) to have good cell coverage, while the other 3 regions (right of the line) showed little or no coverage. Note that this image has not been post-processed and so does not capture the detail that is apparent under live inspection. This area was then illuminated with 20 mW/cm^2^ light and changes in the LFP were recorded. Again, in areas of no cell coverage (Chs. 5, 6, and 7), no response or change in the LFP was seen. Conversely, an average response of ΔV~100 μV (steady state, at the given dose) was seen in areas with coax electrodes in sufficient contact with cells to record LFPs (Chs. 1–4), showing a direct correspondence with the cell coverage observed from fluorescence microscopy, Figure 6B. Similar results were found in the three other cMEA devices.

### Through-coax optical excitation

**Figure 7:**
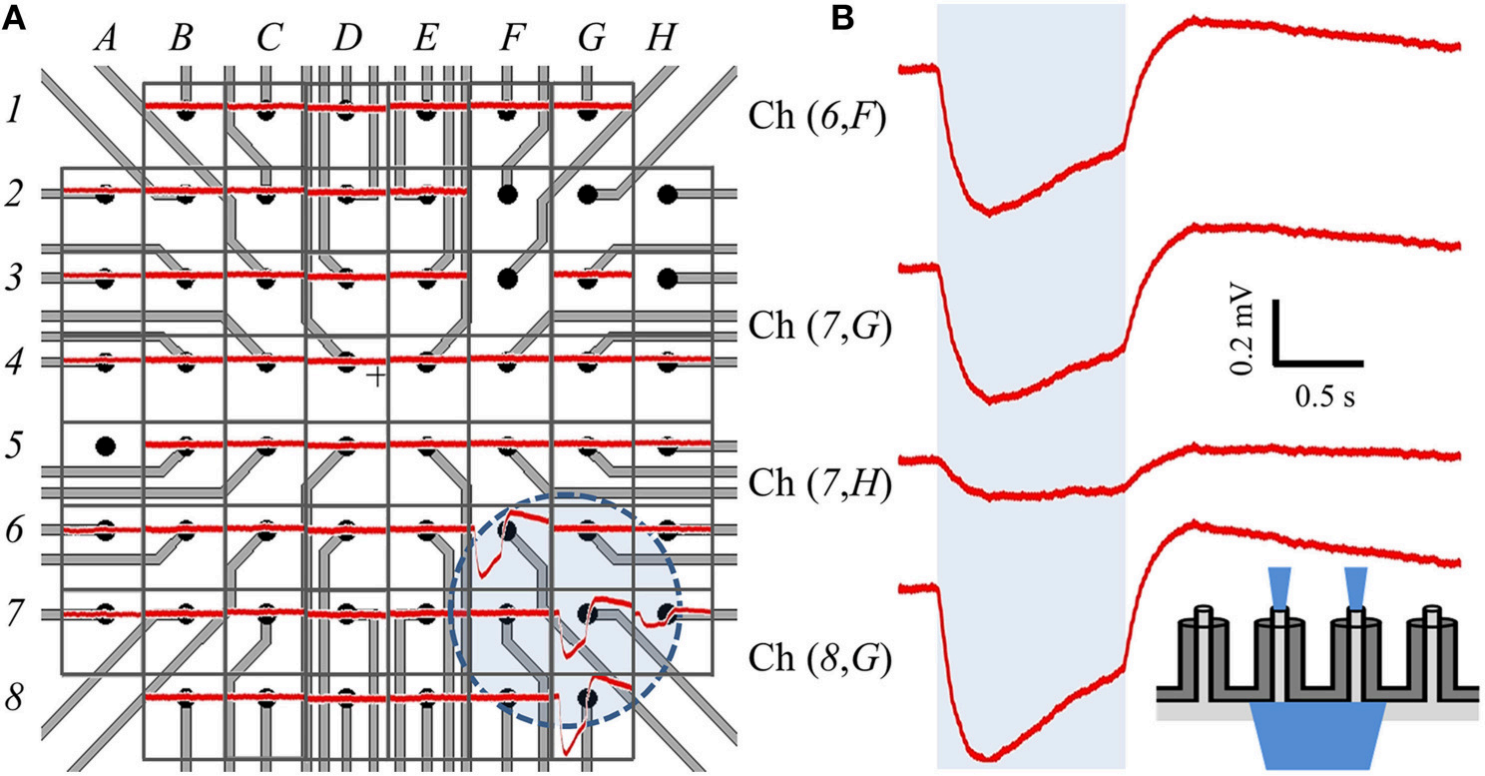
**Backside stimulation of HEK-ChR2 cells cultured on cMEA. (A)** Layout of cMEA chip having 60 coaxial sensing regions, with recorded data overlain. The sensing regions are 20 μm in diameter at 100 μm pitch. The (*number, letter*) combinations correspond to (row, column) recording channels. The voltage response to optical illumination (at 473 nm) for each region is plotted in red. Regions without data curves had non-working inputs on the measurement amplifier. The shaded circle in the lower right centered near (*7,G*) indicates the illuminated area for this particular experiment. **(B)** Expanded views of signals from four regions within illuminated area, showing clear voltage deflections due to optical stimulus. Shaded region represents light-on times. Inset depicts light-from-below configuration. That is, light is input from below the array, passes through the coax cores, and stimulates cells above the array.

We modified the orientation of our optical source to be incident on the backside of a different cMEA, which now contained 60 coaxial sensing regions, 20 μm in diameter at 100 μm pitch. Through-coax optical excitation was achieved by fabricating the cMEA such that the substrate was opaque everywhere except through the coax cores (see inset of Figure 7B for schematic). Initial recordings of the device in cell culture medium alone (i.e., without cells) were made to establish a baseline noise level, and to determine and record photoelectric artifacts induced by the laser, should any occur, for the purpose of post-data analysis filtering. However, no optical artifacts were seen throughout these initial measurements. As above, HEK-ChR2 cells were grown on the device and coverage was confirmed by microscopy. We next positioned the optical fiber below each of the sensing regions and performed a dose-response test at any site with an event. Once again, the locations of detected events on the cMEA corresponded directly to the locations of the laser and were roughly confined to the extent of the spot size, as shown by the circle in Figure 7A and the corresponding LFP responses in Figure 7B.

## Discussion

In this report on the development of a neuroelectronic device architecture based on micro- and nanocoaxial arrays with optogenetic applications, our devices demonstrated extracellular sensing of biological perturbations in the LFP while also having the capability to localize a stimulating light source. Devices fabricated on the scale of traditional MEAs were shown to have sensitivity (i.e., signal-to-noise ratio 5:1 or higher) comparable to extant devices (Spira and Hai, 2013). Furthermore, our modeling has shown that local shielding minimizes crosstalk between adjacent pixels, as in Figure 1, suggesting that future devices may resolve extracellular events on increasingly smaller scales than are currently resolvable with high-density unshielded devices.

The ultimate goal of any MEA technology is to record from networks of cells and analyze their circuit dynamics in an effort to provide insight into physiological behavior. To this end, high-density MEAs utilizing complementary metal oxide semiconductor (CMOS) technology have greatly increased the number of recording sites on a single device (Rodger et al., 2008; Huys et al., 2012; Bakkum et al., 2013; Ballini et al., 2014). However, signals generated from electrogenic cells have been shown to spread beyond 100 μm, which presents a problem as unshielded electrodes will have overlapping sensing regions, as reported in Buzsáki (2004), Kajikawa and Schroeder (2011). Traditional spike sorting methods (principal component analysis, wavelet transform, *en bloc*, etc.) require high computational demand and become unreliable due to waveform variability, small spike amplitude and synchronous firing events (Einevoll et al., 2012). Implementing the coaxial architecture to high density arrays represents an alternative way to obtain high density network recording while at the same time suppressing electrical crosstalk.

In addition to minimizing crosstalk, the fact that propagation of light through specific coax regions caused large LFPs from HEK-ChR2 cells demonstrates the ability of the present architecture to facilitate localization of the stimulating light source to the electrode sensor, which is the basis of the optrode. The localization of applied light is also important when using minimum light intensities to mediate the behavior of a particular cell type, as light incident from above the neural assembly will scatter and attenuate upon entering the medium prior to being absorbed by the light-sensitive opsins. Ozden et al. (2013) have previously shown peak intensity to be inversely proportional to stimulating optical fiber aperture diameter, and since the individual coaxes are capable of being fabricated at sub-cellular dimensions (Merlo et al., 2014) (~1 μm), the cNEA could provide a solution for lower power consumption as well as facilitating direct stimulation of an individual cell (or region within a cell). In contrast, when using macroscale optical fibers for such stimulation, the technical problems of tissue damage and unintentional illumination of distal neurons are unavoidable (Buzsáki et al., 2015). Furthermore, the increased distance from the cell in the fiber case necessitates a higher input power, which can cause undesired artifacts. Our device detected a change in the LFP using as little as 0.5 mW/cm^2^ light intensity, something that could be achievable with micro-light-emitting diodes (μLED). It is also worth pointing out that the present data indicate that the cMEA detects field potentials without suffering signal contamination due to artifacts of photostimulation which may occur in semiconductor-based MEAs. The current results thus encourage future study of this device architecture and materials.

A logical next step is the direct incorporation of μLED technology. This might help achieve the attractive technical goal of a self-regulating, closed-loop optogenetic device (Zhang et al., 2009; Anikeeva et al., 2012; Kim et al., 2013; Ozden et al., 2013; Wu et al., 2015), that fully integrates optical and electronic elements in the most compact package. Our fabrication process for the coaxial optrode array lends itself to adjustment of structural parameters (core diameter, core tip sharpness, pillar height, array pitch, flexible substrate, *etc*.) (Rizal et al., 2015) to achieve an optimal shielded optrode array architecture. Thus, these results provide compelling justification for researchers to investigate and further characterize coaxial electrodes in next generation neural interfaces.

## Author contributions

MN conceived of the project. JN, JC, and MN designed the experiments. TCC, JL, and JN prepared the EYFP-expressing HEK-293 cells. JN, JV, TC, JL, JC, and MN performed the experiments. All the authors contributed to the theoretical and experimental analysis of the results. JN, TCC, MB, JC, and MN wrote the paper.

## Funding

Support for this research included grants from the W.M. Keck Foundation to MN, the National Institutes of Health (MH093412) and Brain and Behavior Research Foundation (NARSAD #19417) to JC and the Boston College Integrated Science Undergraduate Research Fellowship to JL.

### Conflict of interest statement

The authors declare that the research was conducted in the absence of any commercial or financial relationships that could be construed as a potential conflict of interest.

## Footnotes

^1^Multichannel Systems MCS GmbH, Reutlingen Germany.
